# Blunt Traumatic Isolated Left External Iliac Vein Injury Without Pelvic Fracture

**DOI:** 10.7759/cureus.104669

**Published:** 2026-03-04

**Authors:** Khuloud H Alnuaimi, Phalguni S Preethi Asapu, Mohamed E Mohamed, Thiagarajan Jaiganesh

**Affiliations:** 1 Emergency Department, Sheikh Tahnoon Bin Mohammed Medical City (STMC), Al-Ain, ARE

**Keywords:** blunt trauma, conservative management, external iliac vein injury, inferior vena cava filter, pelvic hematoma, vascular trauma, venous injury

## Abstract

Isolated iliac vein injuries from blunt trauma are uncommon but potentially life‑threatening. We report a case of isolated left external iliac vein injury in a 39-year-old female patient following blunt trauma sustained in an electric scooter-related motor vehicle collision. Despite the absence of pelvic fractures, the patient presented with hypotension requiring massive hemorrhage protocol activation and vasopressor support. Computed tomography demonstrated a pelvic hematoma with active venous contrast extravasation. Diagnostic angiography was performed, but endovascular repair was not feasible due to the inability to advance the catheter through extensive venous thrombosis. The patient was subsequently managed conservatively with therapeutic anticoagulation initiated on hospital day 5, inferior vena cava (IVC) filter placement for pulmonary embolism prophylaxis given the contraindication to immediate anticoagulation, and supportive care, resulting in hemodynamic stabilization and clinical improvement. This case highlights that isolated iliac vein injuries can occur without pelvic fractures and emphasizes the need for early computed tomography angiography in hemodynamically unstable trauma patients with pelvic hematomas. When endovascular intervention is not technically feasible, conservative management with individualized timing of anticoagulation and selective IVC filter placement can achieve favourable outcomes in appropriately selected patients.

## Introduction

Isolated iliac vein injuries from blunt trauma are uncommon, accounting for approximately 1.2% of all abdominal trauma cases, with the majority occurring in association with pelvic fractures [1]. Analysis of the National Trauma Data Bank demonstrates that isolated iliac vein injuries carry a 30-day mortality rate of 16.5%, significantly lower than the 39.0% mortality observed in patients with non-isolated iliac vascular injuries, but still representing a life-threatening condition [1]. Notably, while most iliac vein injuries occur with pelvic fractures, isolated injuries without skeletal trauma have been reported, though they remain exceedingly rare [2].

The biomechanical mechanisms underlying iliac vein injury in the absence of pelvic fractures are poorly understood but likely involve high-energy deceleration forces, direct compression, or shearing mechanisms that disrupt the vessel wall without causing adjacent bony injury [3,4]. The external iliac vein, despite its anatomically protected pelvic position, may be vulnerable to stretching or compression during rapid deceleration, particularly in motor-vehicle collisions [2]. Unlike arterial injuries, which typically present with rapid hemodynamic deterioration, venous injuries may manifest more subtly with unexplained hypotension, deep pelvic hematomas, or lower-extremity swelling [3]. This diagnostic challenge is compounded by the fact that venous bleeding may be under-recognized on initial imaging, as standard trauma CT protocols are optimized primarily for arterial and solid-organ injuries [3,5].

Contrast-enhanced computed tomography (CT) plays a pivotal diagnostic role by identifying vascular displacement, intrapelvic fluid collections, and active contrast extravasation suggestive of venous bleeding [4,5]. However, venous injuries may be more difficult to detect than arterial injuries, and a high index of suspicion is required in hemodynamically unstable patients with pelvic hematomas but no fractures [3,4].

Management of iliac vein injuries depends on the extent of hemorrhage, vascular accessibility, and the patient's hemodynamic status. Surgical repair remains the preferred treatment when feasible, as ligation has been associated with higher mortality (odds ratio 2.2) compared with repair in isolated iliac vein injuries [1]. Endovascular techniques, including covered-stent placement, are increasingly utilized as less invasive alternatives and have demonstrated favorable outcomes in selected patients [6]. Recent registry data have shown lower mortality rates with endovascular intervention (18%) compared with open surgery (26%) in patients with iliac vessel injuries [6]. However, technical challenges-including extensive thrombosis, limited catheter access, or venous collapse-may preclude successful endovascular intervention [7,8].

In hemodynamically stable patients where intervention is not technically feasible, conservative management with anticoagulation, thromboprophylaxis, and close clinical monitoring may yield favorable outcomes, particularly within a multidisciplinary care framework [6,8].

This case adds to the limited literature on isolated external iliac vein injuries following blunt trauma without pelvic fractures and emphasizes the importance of maintaining a high index of suspicion for venous injury even in the absence of skeletal trauma. It also highlights the role of individualized management strategies when standard endovascular or surgical options are not feasible.

## Case presentation

In May 2025, a 39-year-old female patient was brought to the Emergency Department approximately 30 minutes after being struck by a motor vehicle while riding an electric scooter. On arrival, a primary trauma assessment was conducted according to Advanced Trauma Life Support (ATLS) principles. The patient was alert and oriented, maintaining her airway independently, with a cervical collar in place. She was able to communicate clearly and provide a history of the incident.

Respiratory examination revealed bilateral equal breath sounds with symmetrical chest expansion and a respiratory rate of 18 breaths per minute. Cardiovascular assessment showed strong peripheral pulses and a heart rate of 82 beats per minute; however, the patient was hypotensive with a blood pressure of 77/55 mm Hg. The presence of hypotension without compensatory tachycardia is a recognized phenomenon in hemorrhagic shock, representing the sympathoinhibitory vasodilation phase (Phase 2) of the hemodynamic response to blood loss, which may occur when central blood volume is reduced by approximately 30% [9].

The patient denied use of beta-blockers or other heart rate-limiting medications. Capillary refill time was approximately three seconds, heart sounds were normal, and core temperature was 36.7°C. Initial laboratory investigations revealed a hemoglobin of 10.2 g/dL, serum lactate of 4.1 mmol/L, and base deficit of -6.2 mEq/L, consistent with tissue hypoperfusion and early hemorrhagic shock. Neurological assessment demonstrated a Glasgow Coma Scale (GCS) score of 15/15, with equal and reactive pupils. Point-of-care glucose levels were within normal limits.

Physical examination revealed multiple superficial abrasions and lacerations with localized pain in the right lower limb. The pelvis was clinically stable on examination, and a pelvic binder was applied as a precautionary measure. An extended focused assessment with sonography for trauma (EFAST) was negative for intra-abdominal free fluid; however, the urinary bladder was not visualized, likely due to an empty bladder at the time of assessment.

Initial resuscitation was initiated using warmed balanced crystalloid solution (lactated Ringer's) via a rapid transfuser, and a 1 g bolus of tranexamic acid was administered, resulting in improvement of blood pressure to 105/83 mm Hg. Although current ATLS 11th edition guidelines and damage-control resuscitation principles emphasize early blood product transfusion and limiting crystalloid administration to less than 3 liters in the first six hours, crystalloid was used as a temporizing measure pending activation of the massive hemorrhage protocol [10,11].

**Figure 1:**
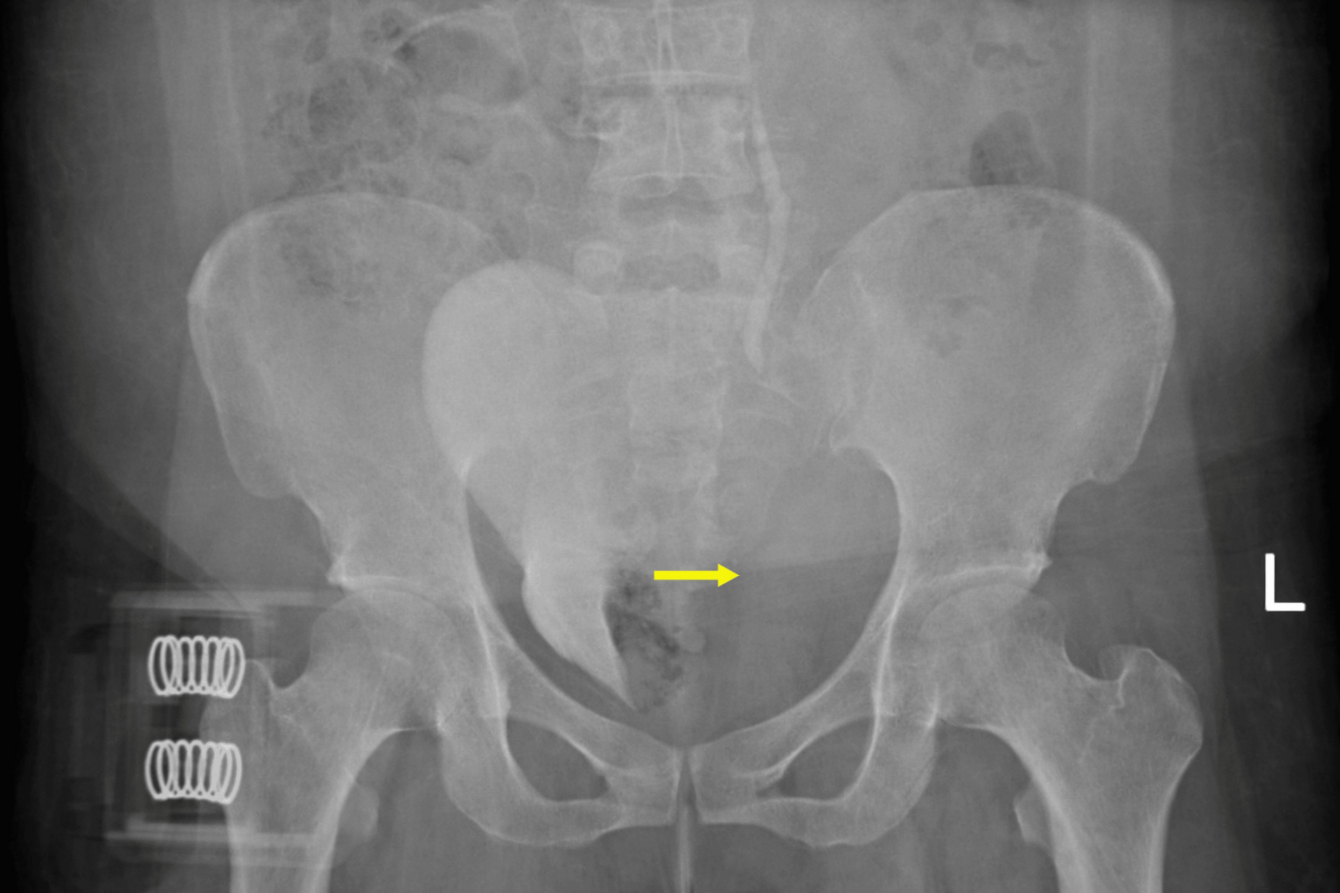
Anteroposterior pelvic radiograph demonstrating rightward displacement of the urinary bladder, suggestive of an adjacent pelvic hematoma. The sacroiliac and hip joints are bilaterally symmetrical, with no evidence of pelvic fracture.

The patient subsequently underwent a comprehensive trauma workup, including whole-body computed tomography (CT), chest and pelvic radiographs, and radiographs of both lower extremities. Chest radiograph and lower limb radiographs were unremarkable. However, the pelvic radiograph demonstrated rightward displacement of the urinary bladder, raising suspicion for an adjacent pelvic hematoma (Figure 1).

**Figure 2:**
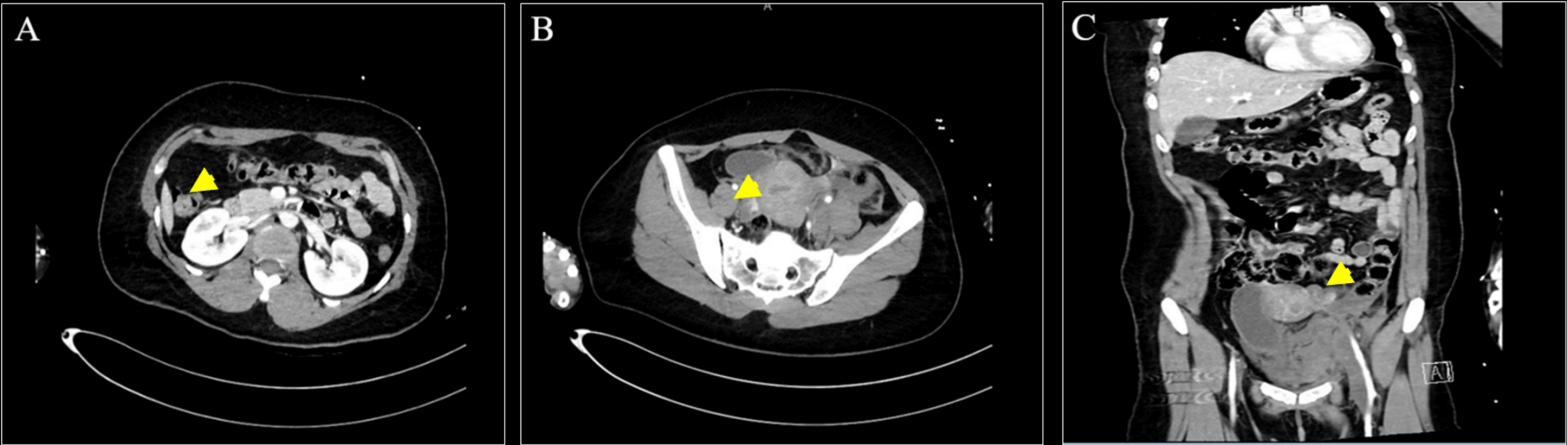
Contrast-enhanced computed tomography of the abdomen and pelvis. (A) Axial pelvic CT demonstrating hyperattenuation adjacent to the left external iliac vessels (arrow) consistent with contrast extravasation. (B) Axial abdominal CT showing extension of hematoma into the left iliopsoas region. (C) Coronal CT reconstruction illustrating the craniocaudal extent of the left-sided retroperitoneal hematoma.

Contrast-enhanced trauma computed tomography (CT) performed with arterial and portal venous phase imaging demonstrated patchy, ill-defined hyperdensity along the left external iliac vessels, consistent with active contrast extravasation. The left external iliac vein was not visualized, raising concern for venous injury and thrombosis. Multiple areas of high attenuation were noted within the left hemipelvis, extending into the left iliopsoas muscle, consistent with a pelvic hematoma. These findings were associated with rightward displacement of the urinary bladder. Prominent surrounding fat stranding and streaks of fluid were present, indicating post-traumatic reactive inflammatory changes (Figure 2).

In light of the imaging findings and the patient's initial hemodynamic instability, the major hemorrhage protocol was activated. She received two units of packed red blood cells, two units of fresh frozen plasma, and one unit of platelets in a balanced ratio. Temporary vasopressor support with norepinephrine was initiated to maintain adequate perfusion pressure until placement of an arterial line, after which blood pressure stabilized at 110/98 mm Hg.

A multidisciplinary trauma team was promptly involved, including General Surgery, Urology, Interventional Radiology (IR), Vascular Surgery, Anesthesiology, and Intensive Care. A two-way urinary catheter was inserted by the urology team, yielding 300 mL of clear urine without hematuria, effectively excluding bladder injury. Vascular Surgery initially recommended conservative management given hemodynamic stabilization with resuscitation. The patient was admitted to the Intensive Care Unit (ICU) for close monitoring while awaiting planned IR evaluation.

**Figure 3:**
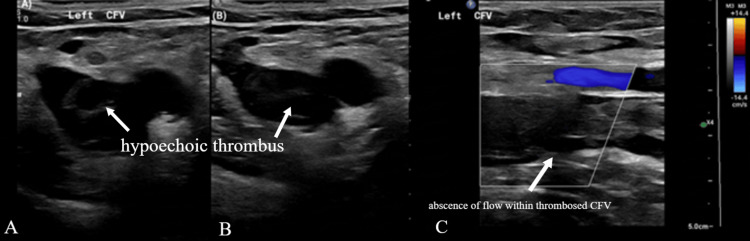
Ultrasound evaluation of the left common femoral vein (CFV). (A–B) Gray‑scale transverse images of the left CFV show a dilated, non‑compressible lumen containing hypoechoic intraluminal material, consistent with acute thrombus. The common femoral artery is seen adjacent for anatomic reference. (C) Color Doppler image demonstrates absence of intraluminal flow within the thrombosed CFV and focal venous displacement by echogenic thrombus, confirming complete venous occlusion due to acute deep venous thrombosis.

Several hours later on the same day, the patient underwent IR-guided vascular evaluation. Ultrasound demonstrated thrombotic occlusion of the left femoral vein (Figure 3).

**Figure 4:**
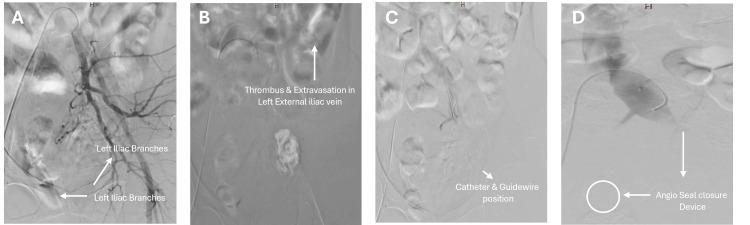
Serial angiography images illustrating diagnostic venography of left external iliac vein thrombotic occlusion. (A) Initial angiogram demonstrating right femoral arterial access and delineation of pelvic vasculature for orientation. (B) Venogram from left femoral micropuncture showing a large thrombus burden with contrast extravasation, confirming left external iliac vein injury. (C) Catheter advancement into the occluded segment with persistent venous obstruction. (D) Final image demonstrating vascular closure using an Angio-Seal (Terumo Medical Corporation, Somerset, NJ, USA) device.

Venous access was first obtained via the right femoral vein using a 5-French sheath; however, attempts to advance a guide catheter into the left external iliac vein were unsuccessful due to extensive thrombosis. A second attempt using a micropuncture technique to access the left femoral vein was successful. Venography revealed a large thrombus with significant contrast extravasation involving the left external iliac vein, consistent with a major venous injury. No endovascular intervention (embolization or stenting) was performed due to the extensive thrombotic occlusion precluding catheter advancement to the site of injury; the procedure was therefore limited to diagnostic angiography only. Concurrent arterial evaluation via right common femoral artery access showed no evidence of active arterial bleeding on left iliac arteriography. Hemostasis at the access site was achieved using an Angio-Seal (Terumo Medical Corporation, Somerset, NJ, USA) closure device (Figure 4).

Following review of the findings, the Vascular Surgery team recommended continuation of conservative management given hemodynamic stability and the self-tamponading effect of the extensive venous thrombosis. During her ICU stay, the patient remained hemodynamically stable and was initiated on mechanical deep vein thrombosis prophylaxis with intermittent pneumatic compression devices. The following day, a temporary inferior vena cava (IVC) filter was placed to prevent pulmonary embolism, given the extensive iliofemoral thrombosis and the initial contraindication to therapeutic anticoagulation due to active hemorrhage, consistent with current guidelines recommending IVC filter placement when anticoagulation is contraindicated in patients with acute DVT [12].

She underwent regular laboratory monitoring, received analgesia for pain control, and continued limb elevation with compression stockings. Transthoracic echocardiography demonstrated normal cardiac function.

**Figure 5:**
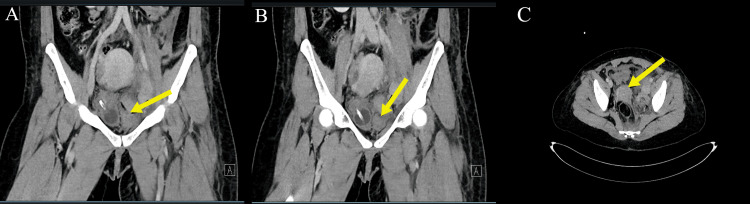
Contrast‑enhanced CT demonstrating left external iliac vein injury. (A–B) Coronal CT images show focal contrast extravasation (arrows) adjacent to the left external iliac vein, indicating active venous bleeding. (C) Axial CT image demonstrates a left‑sided pelvic hematoma (arrow) compressing the external iliac vein, consistent with venous injury without associated pelvic fracture.

Repeat contrast-enhanced CT imaging of the pelvis showed a well-opacified aorta and iliac arteries, with persistent poor visualization of the left external iliac vein from the level of the left common iliac vein bifurcation, likely secondary to surrounding hematoma. The mid-course of the vein appeared distended with central hypodensity, and a small volume of free pelvic fluid was noted (Figure 5).

After four days in the ICU, the patient was transferred to the general surgical ward. She was started on subcutaneous unfractionated heparin at a dose of 5,000 IU three times daily for venous thromboembolism prophylaxis, as therapeutic anticoagulation was initially withheld due to concern for ongoing hemorrhage [13].

One day later, care was transferred to the Vascular Surgery service. With serial hemoglobin levels remaining stable and no clinical or imaging evidence of ongoing bleeding, anticoagulation was escalated to a continuous intravenous heparin infusion at therapeutic doses to treat the established iliofemoral DVT and reduce the risk of thrombus propagation and pulmonary embolism [14]. Serial laboratory investigations confirmed stable hemoglobin levels with progressive clinical improvement.

On hospital day eight, the patient was discharged in stable condition on oral rivaroxaban 15 mg twice daily for treatment of iliofemoral DVT, with planned transition to 20 mg once daily after 21 days and continuation for a minimum of three months. She was instructed to continue compression stockings and analgesics as needed [15]. Follow-up duplex ultrasonography was planned at four to six weeks to assess thrombus resolution and guide IVC filter retrieval. Follow-up with the Vascular Surgery clinic was arranged one week after discharge.

Follow‑up

Despite compliance with compression therapy, she experienced progressive left lower limb pain and edema. One‑month ultrasound showed partial recanalization of the left iliofemoral DVT with predominantly low‑echogenic thrombus; the right iliac veins and IVC were patent with the filter in place. The IVC filter was removed at three months. No further follow‑up has been documented.

The clinical timeline is summarized in Table 1.

**Table 1:** Clinical timeline BID: twice daily; DVT: deep vein thrombosis; ICU: intensive care unit; IV: intravenous; IVC: inferior vena cava; IU: international units; MHP: massive hemorrhage protocol; TID: three times daily; UFH: unfractionated heparin

Day	Events
Day 1	Admission, primary survey, crystalloid resuscitation, MHP activation, vasopressor support, ICU admission, diagnostic angiography performed; endovascular repair not feasible due to extensive thrombosis
Day 2	Temporary IVC filter placement; mechanical DVT prophylaxis initiated
Day 5	Transfer to general surgical ward; prophylactic UFH (5,000 IU TID) initiated
Day 6	Escalation to therapeutic IV heparin infusion
Day 8	Discharge on rivaroxaban 15 mg BID; follow-up arranged

## Discussion

Pelvic venous injuries resulting from blunt trauma are rare and often overshadowed by associated arterial injuries or pelvic fractures. A 2026 National Trauma Data Bank analysis of 2,300 patients with iliac vessel injuries found that the majority (68.9%) resulted from blunt trauma, with treatment modalities including endovascular procedures (41.9%), open surgery (25.1%), and non-operative management (33.0%) [6]. Endovascular treatment was associated with lower mortality rates (18%) compared to open surgery (26%) and non-operative management (19.4%) [6]. A 2024 Trauma Quality Improvement Program analysis of 2,642 patients with iliac and femoral vein injuries reported venous thromboembolism in 10.8% and amputation in 4.2%, with venous ligation identified as the only modifiable independent predictor of amputation [8]. Patients with blunt pelvic vascular injuries have a higher risk of mortality (OR 4.08) and adverse discharge compared to penetrating injuries [16].

The pathophysiology of isolated iliac vein injury in the absence of pelvic fracture likely involves deceleration forces causing shearing or compression of the vessel against the bony pelvis. Unlike arterial hemorrhage, venous bleeding may progress less rapidly due to lower intravascular pressure, allowing for consideration of non-operative management in hemodynamically stable patients. In this case, contrast-enhanced CT findings-including pelvic hematoma, venous displacement, and contrast extravasation-were critical for early diagnosis, consistent with established imaging protocols for blunt abdominopelvic vascular trauma [3-5].

Management of iliac vein injuries remains controversial. Historically, surgical repair has been preferred when feasible, as ligation is associated with higher mortality. A National Trauma Data Bank analysis (2007-2012) demonstrated that ligation of isolated iliac vein injuries had an odds ratio of 2.2 for mortality compared with repair (95% CI, 1.08-4.66), although rates of deep venous thrombosis, pulmonary embolism, fasciotomy, amputation, and acute kidney injury were not significantly different between treatment groups [1]. However, a 2024 Trauma Quality Improvement Program analysis found that venous ligation was the only modifiable independent predictor of amputation, while venous restoration was not an independent predictor of venous thromboembolism [8]. These findings support the principle that repair of iliac vein injuries is preferable to ligation whenever feasible.

Endovascular interventions have emerged as minimally invasive alternatives with favorable outcomes. The 2026 National Trauma Data Bank analysis demonstrated that endovascular intervention was associated with significantly lower odds of in-hospital mortality compared with open vascular treatment in penetrating trauma (adjusted OR 0.067, p < 0.001), though this difference was not statistically significant in blunt trauma (p > 0.05) [6]. Data from the American Association for the Surgery of Trauma (AAST) Prospective Observational Vascular Injury Treatment (PROOVIT) registry showed that endovascular treatment for junctional injuries (including iliac vessels) increased by 5% per year from 2013 to 2019, with endovascular repair associated with non-statistically significant lower mortality (19% vs 29%; p = 0.099) despite higher injury severity scores compared with open repair [17]. Covered stent-graft placement has been successfully used for traumatic iliac vein laceration, with rapid hemodynamic stabilization and sustained patency at 12-month follow-up [7]. However, extensive venous thrombosis, as encountered in our patient, may preclude guidewire advancement or safe device deployment, limiting the role of endovascular therapy.

The decision to place an inferior vena cava (IVC) filter in our patient warrants discussion in light of current evidence. The Western Trauma Association guidelines recommend that IVC filters may be considered in the setting of proximal DVT or PE when therapeutic anticoagulation is contraindicated, but prophylactic placement is not recommended [13]. A landmark multicenter randomized controlled trial (NEJM, 2019) demonstrated that early prophylactic IVC filter placement did not reduce the incidence of symptomatic PE or death at 90 days compared to no filter (13.9% vs 14.4%; HR 0.99) [18]. However, among patients who could not receive prophylactic anticoagulation within 7 days, none in the filter group developed symptomatic PE compared to 14.7% in the control group [18]. The Society of Interventional Radiology recommends against routine prophylactic IVC filter placement in trauma patients without known VTE, noting that routine placement is associated with increased DVT rates [12]. In our patient, the IVC filter was placed due to documented iliac vein thrombosis with active extravasation and initial contraindication to therapeutic anticoagulation, aligning with guideline recommendations for patients with established proximal DVT who cannot receive adequate anticoagulation.

The risk of post-thrombotic syndrome (PTS) is a significant long-term concern following iliofemoral DVT. A 2026 systematic review and meta-analysis estimated PTS risk at 51.1% (95% CI: 39.5-62.7%) for iliofemoral DVT, compared to 30.7% for femoropopliteal and 22.8% for distal DVT [19]. The American Heart Association's 2025 scientific statement emphasizes that patients with iliofemoral DVT represent a high-risk subgroup, with approximately 50% developing any PTS, up to 25% developing moderate-to-severe PTS, and up to 10% developing severe PTS including venous ulceration [20]. The statement recommends that patients with acute iliofemoral DVT should be carefully evaluated and closely monitored, with rapid achievement of therapeutic anticoagulation as the cornerstone of effective therapy [20]. Long-term follow-up with compression therapy, lifestyle modifications, and monitoring for PTS symptoms is therefore essential in this patient population.

Anticoagulation was initiated on hospital day five in our patient, following confirmation of hemodynamic stability and absence of ongoing hemorrhage. This approach aligns with recent multicenter trauma literature suggesting that delayed initiation may reduce hemorrhagic complications in patients with significant bleeding risk [21]. The choice of rivaroxaban for discharge anticoagulation is supported by emerging evidence suggesting potential benefits for PTS prevention compared to vitamin K antagonists [20]. Current guidelines recommend rivaroxaban 15 mg twice daily for 21 days followed by 20 mg once daily, with a minimum treatment duration of three months for provoked DVT [15].

This case highlights several key principles: (1) isolated iliac vein injury should be considered in blunt trauma patients presenting with unexplained hypotension and pelvic hematoma, even without skeletal injury; (2) contrast-enhanced CT with arterial and venous phase imaging is essential for early diagnosis; (3) endovascular intervention may be limited by extensive thrombosis; (4) conservative management with multidisciplinary oversight can achieve favorable outcomes; and (5) IVC filter placement should be reserved for patients with documented VTE who cannot receive adequate anticoagulation, rather than for routine prophylaxis.

## Conclusions

This case highlights that isolated external iliac vein injury may present without pelvic fractures and requires early CT evaluation in unstable trauma patients. When endovascular treatment is not possible, carefully tailored conservative management-including delayed anticoagulation and temporary IVC filtration-can achieve stabilization. Close follow‑up remains essential due to the risk of post‑thrombotic sequelae.
